# Morbidity profile among older people at primary health care centers in Saudi Arabia during the period 2012-2020

**DOI:** 10.15537/smj.2023.44.1.20220465

**Published:** 2023-01

**Authors:** Maysoon M. Al-Amoud, Doaa I. Omar, Eman N. Almashjary, Shaker A. Alomary

**Affiliations:** *From the General Directorate of Health Programs and Chronic Diseases (Al-Amoud, Omar, Almashjary, Alomary), Ministry of Health, Riyadh, Kingdom of Saudi Arabia, and from the Department of Community (Omar), Environmental and Occupational Medicine, Faculty of Medicine, Benha University, Benha, Egypt.*

**Keywords:** older people, primary health care, geriatric giants, morbidity profile

## Abstract

**Objectives:**

To evaluate the morbidity profile and explore the geriatric giants, health problems, and their risk factors among old people in the older people health clinics at primary health care centers (PHCCs) in Saudi Arabia.

**Methods:**

This is a record-based descriptive cross-sectional study. Data was collected between 2012-2020 using the health data of older people to whom comprehensive geriatric assessment (CGA) was carried out at 1,481 PHCCs in Saudi Arabia. Data included sociodemographic and health related characteristics, medications, results of CGA, complete clinical examination, and laboratory results. Assessment was carried out for diabetes, hypertension, obesity, underweight, vision and hearing impairments, depression, memory and cognitive impairment, risk of falls, urine incontinence, bronchial asthma, and anemia.

**Results:**

A total of 193,715 older people were screened. A high prevalence of diabetes (55.4%), hypertension (49.1%), diabetes and hypertension co-morbidity (26.8%), and obesity (22.2%) were found. The overall prevalence of anemia was 4.7% and asthma 8.9%. The prevalence of positive screening for depression was 5.9%, 2.9% for memory and cognitive impairment, 6.3% for urine incontinence, and 4.0% for risk of fall. The prevalence of vision impairment was 20.6%, hearing impairments was 12.6%, and for underweight it was 5.4%. There was high prevalence of risk factors like smoking (8.5%), and polypharmacy (25.3%). Health regions varied widely in prevalence of the studied health conditions.

**Conclusion:**

The study findings highlight the importance of CGA in early detection of geriatric giants, health problems, and associated risk factors among Saudi older people.

**T**he world’s population is aging rapidly alongside broader social and economic changes taking place throughout the world.^1^ Globally, the population aged 60 years or over is estimated to be one in 6 people by 2030, and will double by 2050 (2.1 billion), with nearly two-thirds of them in low- and middle-income countries.^2^

Saudi Arabia is also witnessing an increase in its aging population mainly due to an increase in life expectancy that has improved from 64.4 years in the 1980s to 74.3 years in the 2000s.^3^ As a result, the elderly population aged 60 and above is projected to increase from 3% in 2010 to 9.5% in 2035 and 18.4% in 2050.^4,5^ As the proportion of older people increases, the prevalence of chronic diseases also rises together with the risk of having 2 or more chronic conditions (multi-morbidity).^6^ Chronic diseases cause medical, social, and psychological problems that limit the activities of elderly people in the community.^7^

Common health conditions among older people include sensory disorders (hearing loss, cataracts, and refractive errors), musculoskeletal disorders (back and neck pain, and osteoarthritis), chronic obstructive pulmonary disease, diabetes, depression, and dementia. Furthermore, as people age, they are more likely to experience geriatric syndromes which refer to several complex health conditions that occur simultaneously and do not fall into discrete disease categories; these are often the consequence of multiple underlying factors and include frailty, urine incontinence, falls, delirium, and pressure ulcers.^2^ The combination of multi-morbidity, age-related frailty, geriatric syndromes, and acute illness places older people at increased risk for adverse outcomes such as long-term dependence, increased demands on costs for health and social care including increased admission to a nursing home, and ultimately death.^8,9^

Comprehensive geriatric assessment (CGA) has been developed in response to the health issues and problems experienced by older people who require hospital-level care that not early discovered and managed, and refers to a multi-dimensional diagnostic and therapeutic process that is focused on determining a frail older person’s medical, functional, mental, and social capabilities and limitations with the goal of ensuring that problems are identified, quantified, and managed appropriately. CGA has the potential to improve health outcomes, reduce the costs of health care and social care, and reduce the caregiver’s burden.^9,10^

In Saudi Arabia, the health services to older people aged 60 years and above are provided at primary health care centers (PHCCs) through the Older People Health Care Program (OPHCP) since 2012, when the program was established. This program is the first step obtained by the Saudi Ministry of Health (MOH) to improve the health services as a response to meet the health demands of the older people and introduce the geriatric health services in MOH health institutes.^11,12^ Since the services are provided at the PHCC level, the main scope of these services is preventive as well as curative for chronic diseases usually managed at PHCCs. The preventive services include CGA, health education to older people and their caregiver, and immunization. The aim of CGA is to detect the common health conditions among older people targeted by the program.^12^

The aim of this study is to evaluate the morbidity profile and explore the geriatric giants, common health problems, and their risk factors among older people in the older people health clinics and to whom CGA was carried out at PHCCs during the period 2012-2020. This will help improve the planning and prioritizing of the health services, resource allocation and appropriate effective interventions for older people.

## Methods

This was a record-based descriptive cross-sectional study using the health data of older people 60 years and above to whom geriatric health services were delivered at the 1,481 PHCCs that implement OPHCP during the period 2012-2020. The total number of older people screened was 193,715.

The health data of older people involved in the OPHCP that were registered in the CGA file for older people were collected annually. These data included sociodemographic characteristics, health-related characteristics, medications, results of CGA, results of complete clinical examination and assessment, and laboratory results.

The CGA was carried out to the target group during the first visit to the PHCC. The components of the CGA were the assessments of the common geriatric conditions recommended by the Wolrd Health Organization (WHO) for age-friendly PHC setting. This included assessment of the 4 geriatric giants (depression, memory impairment, risk of falls, and urine incontinence), vision and hearing impairments, and 2 common chronic conditions (diabetes [DM] and hypertension [HTN]).^13^ In addition, bronchial asthma, obesity, underweight, and anemia were assessed. The assessment was carried out by trained doctors and nurses.

The final results of the CGA of the studied group were recorded in the CGA files of the older people, then routinely collected from the PHCCs by filling a data collection sheet, then reported periodically to the OPHCP head office for analysis, and then reported to the higher authorities to provide the necessary appropriate feedback or action to the health region.^12^ This study was approved by the research ethics committee at the Central Institutional Review Board at MOH, Riyadh, Saudi Arabia (approval number: 22-26 M).

The different tools used for the assessment of the geriatric population included: I) Screening tool for depression; the OPHCP utilized the 15-item geriatric depression scale (GDS-15) to screen older people for depression.^13^ II) Screening tool for memory and cognitive impairment: the OPHCP utilized the Mini-Cog test to screen older people. The MOH obtained permission to utilize the English and Arabic versions of the test in 2014.^12,14,15^ This was taken into consideration during data analysis of cognitive impairment where the percentage of the cognitive impairment (total and for each region) was calculated for the total older people to whom Mini-Cog test was carried out from 2014-2021. III) For screening risk of fall, a multi-factorial risk assessment was carried out along with history of fall if any. Gait assessment Up and Go, and Romberg’s balance tests were used.^13,16^ IV) Urine incontinence among the studied group was assessed by history taking, medication review, and investigations.^13^ V) Special sense assessment of the participants: assessment of visual acuity was carried out by history taking and Snellen’s chart. Assessment of hearing acuity was carried out by history taking and whispering test.^13^ VI) The body mass index (BMI) ([weight in kg/(height in m^2^)]) was calculated for each participant to determine obesity (BMI≥30 kg/m^2^) and underweight (BMI<18.5 kg/m^2^ based on the WHO international standard.^17^ VII) Anemia was diagnosed by using WHO criteria for anemia (hemoglobin of less than 13 g/dL in men and less than 12 g/dL in women).^18^

### Statistical analysis

Data were analyzed using the Statistical Package for Social Sciences, version 22.0 (IBM Corp., Armonk, NY, USA). Microsoft Excel 2016 MSO (version 2209) was also used. Categorical variables were expressed as percentages. Statistical tests of significance were not carried out.

## Results

The total number of screened older people during the period from 2012-2020 was 193,715. Most of these participants (39.5%) belonged to the age group 60-64 years, and only 7.9% were above 80 years. Females represented 51.5% of the participants, 64.5% of participants were illiterate, while only 2.5% were highly educated, and 7.7% were working. The percentage of older people caring for themselves was 51.5%, while 37.5% of them were cared for by a family member. Walking aids were used by 21.9% while 4.7% were using a wheelchair (Table 1).

**Table 1:** - Sociodemographic characteristics of the studied group during the period 2012-2020.

Variables	Male	Female	Total
***Studied group***			
2012	3113 (46.8)	3543 (53.2)	6656 (3.4)
2013	6393 (48.8)	6717 (51.2)	13110 (6.8)
2014	7788 (47.7)	8543 (42.3)	16331 (8.4)
2015	9516 (47.4)	10573 (52.6)	20089 (10.4)
2016	10840 (48.9)	11307 (51.1)	22147 (11.4)
2017	12830 (49.0)	13368 (51.0)	26198 (13.5)
2018	12524 (46.0)	14711 54.0)	27235 14.1)
2019	12905 (47.7)	14119 (52.2)	27024 (14.0)
2020	17977 (51.5)	16948 (48.5)	34925 (18.0)
Total	93886 (48.5)	99829 (51.5)	193715 (100)
***Age groups***			
60-64 years	36275 (38.6)	40183 (40.3)	76458 (39.5)
65-74 years	30034 (32.0)	34124 (34.2)	64158 (33.1)
75-84 years	19629 (20.9)	18112 (18.1)	37741 (19.5)
+85 years	7948 (8.5)	7410 (7.4)	15358 (7.9)
***Education***			
Illiterate	52703 (56.4)	71374 (72.1)	124077 (64.5)
Primary education	23714 (25.4)	18699 (18.9)	42413 (22.0)
Preparatory and sec	13794 (14.7)	7459 (7.5)	21253 (11.0)
Faculty or higher	3314 (3.5)	1473 (1.5)	4787 (2.5)
***Working status***			
Currently working	11751 (12.6)	3094 (3.1)	14845 (7.7)
***Type of caregiver***			
Self-care	49509 (53.3)	49162 (49.8)	98671 (51.5)
Family member	34087 (36.7)	37831 (38.3)	71918 (37.5)
Paid caregiver	9297 (10.0)	11791 (11.9)	21088 (11.0)
***Functional status***			
Walking w/o any aids	65149 (73.3)	69755 (73.4)	134904 (73.4)
Walking with aids	19629 (22.1)	20636 (21.7)	40265 (21.9)
On wheelchair	4068 (4.6)	4661 (4.9)	8729 (4.7)

Values are presented as a number and precentage (%). w/o: without, sec: secondary

Table 2 illustrates the region-wise distribution of the investigated chronic diseases and conditions among the studied group during the period 2012-2020. The overall prevalence of clinically diagnosed DM was 55.4%, and it was higher among males (56.1%) than among females (54.7%). The overall prevalence of HTN was 49.1%, and the prevalence among females (50%) was higher than among males (48.1%). The screened older people with both DM and HTN was 26.8%, with a higher prevalence among females (27.6%) than among males (25.9%). The overall prevalence of obesity was 22.2%, and it is higher among females (24.9%) than males (19.4%). The overall prevalence of underweight was 5.4%, and it is higher among males (5.6%) than females (5.2%). The overall prevalence of anemia was 4.7% and it was higher among females (5.5%) than males (3.9%). Asthma was reported in 8.9% of the total participants, and it was higher among females (8.9%) than males (8.8%).

**Table 2:** - Region-wise distribution of some chronic diseases and conditions among the studied group during the period 2012-2020.

Health regions	All screened	Diabetes			Hypertension			DM & HTN			Obesity		
		Male	Female	Total	Male	Female	Total	Male	Female	Total	Male	Female	Total
Asir	27643	6412	5586	11998	5396	5051	10447	2797	2494	5291	2429	2877	5306
	(14.3)	(44.6)	(42.1)	(43.4)	(37.6)	(38.1)	(37.8)	(19.5)	(18.8)	(19.1)	(16.9)	(21.7)	(19.2)
Gassim	25354	8108	7578	15686	7259	6736	13995	3835	3758	7593	2404	3201	5605
	(13.1)	(60.3)	(63.6)	(61.9)	(54.0)	(56.6)	(55.2)	(28.5)	(31.6)	(29.9)	(17.9)	(26.9)	(22.1)
Baha	25450	7199	7194	14393	5783	6801	12584	3117	3684	6801	3085	4186	7271
	(13.1	(57.5)	(55.7)	(56.6)	(46.2)	(52.6)	(49.4)	(24.9)	(28.5)	(26.7)	(24.6)	(32.4)	(28.6)
Jazan	21281	4351	4974	9325	4068	5120	9188	1963	2551	4514	1502	2131	3633
	(11.0)	(47.8)	(40.9)	(43.8)	(44.7)	(42.0)	(43.2)	(21.6)	(21.0)	(21.2)	(16.5)	(17.5)	(17.1)
Riyadh	20667	5907	5853	11760	5544	5501	11045	2702	2767	5469	2038	2558	4596
	(10.7)	(57.1)	(56.7)	(56.9)	(53.6)	(53.3)	(53.4)	(26.1)	(26.8)	(26.5)	(19.7)	(24.8)	(22.2)
Eastern region	12588	3696	3918	7614	3064	3814	6878	1985	2371	4356	1087	1506	2593
	(6.5)	(60.5)	(60.5)	(60.5)	(50.1)	(58.9)	(54.6)	(32.5)	(36.6)	(34.6)	(17.8)	(23.3)	(20.6)
Hafer Albatin	7992	2566	2594	5160	1802	1793	3595	839	867	1706	585	863	1448
	(4.1)	(77.8)	(55.3)	(64.6)	(54.6)	(38.2)	(45.0)	(25.4)	(18.5)	(21.3)	(17.7)	(18.4)	(18.1)
Jeddah	7748	2769	2293	5062	2598	1889	4487	1732	1351	3083	1197	1473	2670
	(4.0)	(63.9)	(67.1)	(65.3)	(60.0)	(55.3)	(57.9)	(40.0)	(39.5)	(39.8)	(27.6)	(43.1)	(34.5)
Hail	6649	1826	2269	4095	1774	2007	3781	998	1146	2144	427	826	1253
	(3.4)	(60.2)	(62.7)	(61.6)	(58.5)	(55.5)	(56.9)	(32.9)	(31.7)	(32.2)	(14.1)	(22.8)	(18.8)
Nagran	5415	1576	1889	3465	1363	1619	2982	642	889	1531	363	700	1063
	(2.8)	(60.1)	(67.6)	(64.0)	(52.0)	(58.0)	(55.1)	(24.5)	(31.8)	(28.3)	(13.8)	(25.1)	(19.6)
Tabuk	4453	1047	1336	2383	986	1321	2307	611	815	1426	331	565	896
	(2.3)	(49.3)	(57.3)	(53.5)	(46.5)	(56.7)	(51.8)	(28.8)	(35.0)	(32.0)	(15.6)	(24.2)	(20.1)
Alahsaa	3921	1421	1241	2662	1421	1397	2818	890	928	1818	549	737	1286
	(2.0)	(69.4)	(66.3)	(67.9)	(69.4)	(74.6)	(71.9)	(43.5)	(49.5)	(46.4)	(26.8)	(39.3)	(32.8)
Almadinah	3696	583	1841	2424	467	1744	2211	284	1173	1457	132	568	700
	(1.9)	(62.5)	(66.6)	(65.6)	(50.1)	(63.1)	(59.8)	(30.4)	(42.5)	(39.4)	(14.1)	(20.6)	(18.9)
Jouf	3678	1161	1045	2206	796	843	1639	472	501	973	634	641	1275
	(1.9)	(60.4)	(59.5)	(60.0)	(41.4)	(48.0)	(44.6)	(24.6)	(28.5)	(26.5)	(33.0)	(36.5)	(34.7)
Konfothah	3627	964	1423	2387	606	1371	1977	321	710	1031	212	449	661
	(1.9)	(65.9)	(65.8)	(65.8)	(41.4)	(63.4)	(54.5)	(21.9)	(32.8)	(28.4)	(14.5)	(20.7)	(18.2)
Makkah	3359	720	728	1448	498	609	1107	257	310	567	201	181	382
	(1.7)	(41.0)	(45.4)	(43.1)	(28.4)	(37.9)	(33.0)	(14.7)	(19.3)	(16.9)	(11.5)	(11.3)	(11.4)
Northern borders	3320	748	1149	1897	657	1125	1782	363	630	993	302	490	792
	(1.7)	(58.0)	(56.6)	(57.1)	(51.0)	(55.4)	(53.7)	(28.2)	(31.0)	(29.9)	(23.4)	(24.1)	(23.9)
Taif	3311	707	915	1622	358	479	837	195	314	509	279	315	594
	(1.7)	(51.5)	(47.2)	(49.0)	(26.1)	(24.7)	(25.3)	(14.2)	(16.2)	(15.4)	(20.3)	(16.3)	(17.9)
Bisha	2297	527	533	1060	524	474	998	249	239	488	232	296	528
	(1.2)	(45.7)	(46.6)	(46.1)	(45.4)	(41.4)	(43.4)	(21.6)	(20.9)	(21.2)	(20.1)	(25.9)	(23.0)
Gurayyat	1266	373	291	664	238	213	451	108	104	212	210	277	487
	(0.7)	(58.4)	(46.4)	(52.4)	(37.2)	(34.0)	(35.6)	(16.9)	(16.6)	(16.7)	(32.9)	(44.2)	(38.5)
Total	193715	52661	54650	107311	45202	49907	95109	24360	27602	51962	18199	24840	43039
	(100)	(56.1)	(54.7)	(55.4)	(48.1)	(50.0)	(49.1)	(25.9)	(27.6)	(26.8)	(19.4)	(24.9)	(22.2)

Health regions	All screened	Underweight			Anemia^*^			Bronchial asthma		
		Male	Female	Total	Male	Female	Total	Male	Female	Total
Asir	27643	705	577	1282	211	262	473	928	833	1761
	(14.3)	(4.9)	(4.3)	(4.6)	(1.5)	(2.0)	(1.7)	(6.5)	(6.3)	(6.4)
Gassim	25354	745	1064	1809	475	661	1136	1237	1240	2477
	(13.1)	(5.5)	(8.9)	(7.1)	(3.5)	(5.6)	(4.5)	(9.2)	(10.4)	(9.8)
Baha	25450	499	513	1012	344	503	847	1067	1287	2354
	(13.1)	(4.0)	(4.0)	(4.0)	(2.7)	(3.9)	(3.3)	(8.5)	(10.0)	(9.2)
Jazan	21281	204	265	469	930	1226	2156	484	596	1080
	(11.0)	(2.2)	(2.2)	(2.2)	(10.2)	(10.1)	(10.1)	(5.3)	(4.9)	(5.1)
Riyadh	20667	617	647	1264	308	575	883	1608	1692	3300
	(10.7)	(6.0)	(6.8)	(6.1)	(3.0)	(5.6)	(4.3)	(15.5)	(16.4)	(16.0)
Eastern region	12588	450	128	578	118	172	290	454	474	928
	(6.5)	(7.4)	(2.0)	(4.6)	(1.9)	(2.7)	(2.3)	(7.4)	(7.3)	(7.4)
Hafer Albatin	7992	4	3	7	8	4	12	184	246	430
	(4.1)	(0.1)	(0.1)	(0.1)	(0.2)	(0.1)	(0.2)	(5.6)	(5.2)	(5.4)
Jeddah	7748	1100	870	1970	265	416	681	362	377	739
	(4.0)	(25.4)	(25.5)	(25.4)	(6.1)	(12.2)	(8.8)	(8.4)	(11.0)	(9.5)
Hail	6649	50	111	161	55	158	213	618	504	1122
	(3.4)	(1.6)	(3.1)	(2.4)	(1.8)	(4.4)	(3.2)	(20.4)	(13.9)	(16.9)
Nagran	5415	101	108	209	37	77	114	192	365	557
	(2.8)	(3.9)	(3.9)	(3.9)	(1.4)	(2.8)	(2.1)	(7.3)	(13.1)	(10.3)
Tabuk	4453	222	272	494	141	236	377	229	159	388
	(2.3)	(10.5)	(11.7)	(11.1)	(6.6)	(10.1)	(8.5)	(10.8)	(6.8)	(8.7)
Alahsaa	3921	100	102	202	197	236	433	111	142	253
	(2.0)	(4.9)	(5.4)	(5.2)	(9.6)	(12.6)	(11.0)	(5.4)	(7.6)	(6.5)
Almadinah	3696	44	73	117	38	119	157	54	157	211
	(1.9)	(4.7)	(2.6)	(3.2)	(4.1)	(4.3)	(4.2)	(5.8)	(5.7)	(5.7)
Jouf	3678	35	58	93	102	130	232	88	85	173
	(1.9)	(1.8)	(3.3)	(2.5)	(5.3)	(7.4)	(6.3)	(4.6)	(4.8)	(4.7)
Konfothah	3627	72	148	220	163	267	430	141	266	407
	(1.9)	(4.9)	(6.8)	(6.1)	(11.1)	(12.3)	(11.9)	(9.6)	(12.3)	(11.2)
Makkah	3359	21	20	41	20	27	47	61	63	124
	(1.7)	(1.2)	(1.2)	(1.2)	(1.1)	(1.7)	(1.4)	(3.5)	(3.9)	(3.7)
Northern borders	3320	30	61	91	52	68	120	45	81	126
	(1.7)	(2.3)	(3.0)	(2.7)	(4.0)	(3.3)	(3.6)	(3.5)	(4.0)	(3.8)
Taif	3311	44	57	101	61	85	146	83	93	176
	(1.7)	(3.2)	(2.9)	(3.1)	(4.4)	(4.4)	(4.4)	(6.0)	(4.8)	(5.3)
Bisha	2297	163	95	258	130	179	309	264	226	490
	(1.2)	(14.1)	(8.3)	(11.2)	(11.3)	(15.6)	(13.5)	(22.9)	(19.8)	(21.3)
Gurayyat	1266	8	17	25	40	41	81	42	39	81
	(0.7)	(1.3)	(2.7)	(2.0)	(6.3)	(6.5)	(6.4)	(6.6)	(6.2)	(6.4)
Total	193715	5214	5189	10403	3695	5442	9137	8252	8925	17177
	(100)	(5.6)	(5.2)	(5.4)	(3.9)	(5.5)	(4.7)	(8.8)	(8.9)	(8.9)

Values are presented as a number and precentage (%). DM: diabetes, HTN: hypertension

Values are presented as a number and precentage (%).

^*^ Males Hb<13g/dl and Females Hb<12g/dl.

In addition, Table 2 illustrates that the prevalence of DM (67.9%), HTN (71.9%), and both DM and HTN (46.4%) co-morbidity were the highest among participants from Alahsaa region. Gurayyat had the highest obesity prevalence (38.5%) while Jeddah showed the highest prevalence of underweight (25.4%). Both anemia (13.5%) and bronchial asthma (21.3%) prevalence were the highest in Bisha.

Table 3 illustrates the region-wise distribution of the investigated geriatric giants among the studied group during the period 2012-2020. The overall prevalence of positive screening for depression was 5.9%, while the overall prevalence of memory and cognitive impairment during the period 2014-2020 was 2.9%. Urine incontinence was reported in 6.3% of the studied participants while 4.0% were at risk of fall. The highest prevalence of depression (18.9%) and risk of fall (24.5%) were found in Jeddah. Alahsaa reported the highest rate of cognitive impairment (10.7%) while the prevalence of urine incontinence was the highest in Konfothah (10.6%). Hafer Albatin reported the lowest prevalence of positive screening for depression (2.1%), cognitive impairment (0.2%), and urine incontinence (2.2%) while Makkah scored the lowest in terms of risk of fall (1.3%).

**Table 3:** - Region-wise distribution of the geriatric giants among the studied group during the period 2012-2020.

Health regions	All Screened	Positive screening for depression			Positive screening for cognitive impairment#			Positive screening for urine incontinence			Positive screening for risk of fall		
		Male	Female	Total	Male	Female	Total	Male	Female	Total	Male	Female	Total
Asir	27643	599	703	1302	321	347	668	541	493	1034	296	359	655
	(14.3)	(4.2)	(5.3)	(4.7)	(2.2)	(2.6)	(2.4)	(3.8)	(3.7)	(3.7)	(2.1)	(2.7)	(2.4)
Gassim	25354	823	697	1520	326	432	758	802	1542	2344	295	383	678
	(13.1)	(6.1)	(5.9)	(6.0)	(2.4)	(3.6)	(3.0)	(6.0)	(13.0)	(9.2)	(2.2)	(3.2)	(2.7)
Baha	25450	363	679	1042	291	350	641	475	680	1155	212	309	521
	(13.1)	(2.9)	(5.3)	(4.1)	(2.3)	(2.7)	(2.5)	(3.8)	(5.3)	(4.5)	(1.7)	(2.4)	(2.0)
Jazan	21281	338	578	916	114	237	351	483	629	1112	257	560	817
	(11.0)	(3.7)	(4.7)	(4.3)	(1.3)	(1.9)	(1.6)	(5.3)	(5.2)	(5.2)	(2.8)	(4.6)	(3.8)
Riyadh	20667	801	961	1762	227	226	453	722	771	1493	207	244	451
	(10.7)	(7.7)	(9.3)	(8.5)	(2.2)	(2.2)	(2.2)	(7.0)	(7.5)	(7.2)	(2.0)	(2.4)	(2.2)
Eastern region	12588	171	275	446	154	164	318	580	633	1213	216	315	531
	(6.5)	(2.8)	(4.2)	(3.5)	(2.5)	(2.5)	(2.5)	(9.5)	(9.8)	(9.6)	(3.5)	(4.9)	(4.2)
Hafer Albatin	7992	63	106	169	8	8	16	91	86	177	264	346	610
	(4.1)	(1.9)	(2.3)	(2.1)	(0.2)	(0.2)	(0.2)	(2.8)	(1.8)	(2.2)	(8.0)	(7.4)	(7.6)
Jeddah	7748	835	627	1462	350	367	717	277	446	723	966	932	1898
	(4.0)	(19.3)	(18.4)	(18.9)	(8.1)	(10.7)	(9.3)	(6.4)	(13.1)	(9.3)	(22.3)	(27.3)	(24.5)
Hail	6649	209	306	515	198	92	290	220	228	448	69	61	130
	(3.4)	(6.9)	(8.5)	(7.7)	(6.5)	(2.5)	(4.4)	(7.3)	(6.3)	(6.7)	(2.3)	(1.7)	(2.0)
Nagran	5415	82	94	176	62	58	120	72	105	177	34	67	101
	(2.8)	(3.1)	(3.4)	(3.3)	(2.4)	(2.1)	(2.2)	(2.7)	((3.8)	(3.3)	(1.3)	(2.4)	(1.9)
Tabuk	4453	77	161	238	34	69	103	154	203	357	36	80	116
	(2.3)	(3.6)	(6.9)	(5.3)	(1.6)	(3.0)	(2.3)	(7.3)	8.7)	(8.0)	(1.7)	(3.4)	(2.6)
Alahsaa	3921	360	276	636	241	179	420	209	149	358	126	118	244
	(2.0)	(17.6)	(14.7)	(16.2)	(11.8)	(9.6)	(10.7)	(10.2)	(8.0)	(9.1)	(6.2)	(6.3)	(6.2)
Almadinah	3696	42	104	146	36	56	92	51	298	349	38	147	185
	(1.9)	(4.5)	(3.8)	(4.0)	(3.9)	(2.0)	(2.5)	(5.5)	(10.8)	(9.4)	(4.1)	(5.3)	(5.0)
Jouf	3678	61	73	134	46	55	101	77	91	168	27	66	93
	(1.9)	(3.2)	(4.2)	(3.6)	(2.4)	(3.1)	(2.7)	(4.0)	(5.2)	(4.6)	(1.4)	(3.8)	(2.5)
Konfothah	3627	66	134	200	60	71	131	131	253	384	97	199	296
	(1.9)	(4.5)	(6.2)	(5.5)	(4.1)	(3.3)	(3.6)	(9.0)	(11.7)	(10.6)	(6.6)	(9.2)	(8.2)
Makkah	3359	59	56	115	18	20	38	79	98	177	14	28	42
	(1.7)	(3.4)	(3.5)	(3.4)	(1.0)	(1.2)	(1.1)	(4.5)	(6.1)	(5.3)	(0.8)	(1.7)	(1.3)
Northern borders	3320	60	102	162	59	25	84	102	138	240	23	57	80
	(1.7)	(4.7)	(5.0)	(4.9)	(4.6)	(1.2)	(2.5)	(7.9)	(6.8)	(7.2)	(1.8)	(2.8)	(2.4)
Taif	3311	109	166	275	44	68	112	73	102	175	42	77	119
	(1.7)	(7.9)	(8.6)	(8.3)	(3.2)	(3.5)	(3.4)	(5.3)	(5.3)	(5.3)	(3.1)	(4.0)	(3.6)
Bisha	2297	83	75	158	43	51	94	52	98	150	31	29	60
	(1.2)	(7.2)	(6.6)	(6.9)	(3.7)	(4.5)	(4.1)	(4.5)	(8.6)	(6.5)	(2.7)	(2.5)	(2.6)
Gurayyat	1266	31	17	48	18	15	33	30	22	52	12	14	26
	(0.7)	(4.9)	(2.7)	(3.8)	(2.8)	(2.4)	(2.6)	(4.7)	(3.5)	(4.1)	(1.9)	(2.2)	(2.1)
Total	193,715	5232	6190	11422	2650	2890	5540	5221	7065	12286	3262	4391	7653
	(100)	(5.6)	(6.2)	(5.9)	(2.8)	(2.9)	(2.9)	(5.6)	(7.1)	(6.3)	(3.5)	(4.4)	(4.0)

Values are presented as a number and precentage (%).

The prevalence of hearing impairment among the studied group was 12.6% while the prevalence of deafness was 1.4%. Visual impairment was found in 20.6% of the screened older persons and 1.1% were blind. Konfothah reported the highest prevalence of hearing (29.7%) and vision impairment (32.7%), Bisha reported the highest prevalence of blindness (3.8%), and Jeddah the highest prevalence of deafness (4.4%). Hafer Albatin ranked the lowest in terms of prevalence of deafness (0.0%), blindness (0.0%) and vision impairment (3.6%), while Makkah reported the lowest hearing impairment prevalence (Table 4).

**Table 4:** - Region-wise distribution of special sense disorders among the studied group during the period 2012-2020.

Health regions	All screened	Deafness			Hearing impairment			Blindness			Vision impairment		
		Male	Female	Total	Male	Female	Total	Male	Female	Total	Male	Female	Total
Asir	27643	128	70	198	1209	974	2183	113	101	219	1992	1704	3696
	(14.3)	(0.9)	(0.5)	(0.7)	(8.4)	(7.3)	(7.9)	(0.8)	(0.8)	(0.8)	(13.9)	(12.8)	(13.4)
Gassim	25354	414	335	749	1625	1383	3008	180	173	353	2678	2804	5482
	(13.1)	(3.1)	(2.8)	(3.0)	(12.1)	(11.6)	(11.9)	(1.3)	(1.5)	(1.4)	(19.9)	(23.5)	(21.6)
Baha	25450	162	185	347	1821	1908	3729	89	89	178	3165	3162	6327
	(13.1)	(1.3)	(1.4)	(1.4)	(14.5)	(14.8)	(14.7)	(0.7)	(0.7)	(0.7)	(25.3)	(24.5)	(24.9)
Jazan	21281	48	113	161	782	1141	1923	42	104	146	1884	2020	3904
	(11.0)	(0.5)	(0.9)	(0.8)	(8.6)	(9.4)	(9.0)	(0.5)	(0.9)	(0.7)	(20.7)	(16.6)	(18.3)
Riyadh	20667	77	85	162	1820	1907	3727	87	111	198	1904	1931	3835
	(10.7)	(0.7)	(0.8)	(0.8)	(17.6)	(18.5)	(18.0)	(0.8)	(1.1)	(1.0)	(18.4)	(18.7)	(18.6)
Eastern region	12588	132	59	191	779	753	1532	223	92	315	1394	1362	2756
	(6.5)	(2.2)	(0.9)	(1.5)	(12.7)	(11.6)	(12.2)	(3.6)	(1.4)	(2.5)	(22.8)	(21.0)	(21.9)
Hafer Albatin	7992	0	0	0	271	285	556	0	0	0	158	129	287
	(4.1)	(0.0)	(0.0)	(0.0)	(8.2)	(6.1)	(7.0)	(0.0)	(0.0)	(0.0)	(4.8)	(2.7)	(3.6)
Jeddah	7748	167	173	340	625	573	1198	28	166	194	1236	1240	2476
	(4.0)	(3.9)	(5.1)	(4.4)	(14.4)	(16.8)	(15.5)	(0.6)	(4.9)	(2.5)	(28.5)	(36.3)	(32.0)
Hail	6649	111	92	203	517	465	982	72	53	125	931	1054	1985
	(3.4)	(3.7)	(2.5)	(3.1)	(17.1)	(12.9)	(14.8)	(2.4)	(1.5)	(1.9)	(30.7)	(29.1)	(29.9)
Nagran	5415	17	11	28	248	269	517	23	23	46	461	526	987
	(2.8)	(0.6)	(0.4)	(0.5)	(9.5)	(9.6)	(9.5)	(0.9)	(0.8)	(0.8)	(17.6)	(18.8)	(18.2)
Tabuk	4453	12	19	31	254	365	619	42	28	70	357	503	860
	(2.3)	(0.6)	(0.8)	(0.7)	(12.0)	(15.7)	(13.9)	(2.0)	(1.2)	(1.6)	(16.8)	(21.6)	(19.3)
Alahsaa	3921	55	23	78	314	196	510	41	33	74	533	488	1021
	(2.0)	(2.7)	(1.2)	(2.0)	(15.3)	(10.5)	(13.0)	(2.0)	(1.8)	(1.9)	(26.0)	(26.1)	(26.0)
Almadinah	3696	11	29	40	183	437	620	4	12	16	332	829	1161
	(1.9)	(1.2)	(1.0)	(1.1)	(19.6)	(15.8)	(16.8)	(0.4)	(0.4)	(0.4)	(35.6)	(30.0)	(31.4)
Jouf	3678	8	13	21	228	232	460	7	18	25	393	367	760
	(1.9)	(0.4)	(0.7)	(0.6)	(11.9)	(13.2)	(12.5)	(0.4)	(1.0)	(0.7)	(20.4)	(20.9)	(20.7)
Konfothah	3627	34	52	86	499	580	1079	9	5	14	476	709	1185
	(1.9)	(2.3)	(2.4)	(2.4)	(34.1)	(26.8)	(29.7)	(0.6)	(0.2)	(0.4)	(32.5)	(32.8)	(32.7)
Makkah	3359	2	3	5	111	104	215	4	6	10	306	327	633
	(1.7)	(0.1)	(0.2)	(0.1)	(6.3)	(6.5)	(6.4)	(0.2)	(0.4)	(0.3)	(17.4)	(20.4)	(18.8)
Northern borders	3320	8	9	17	210	256	466	6	5	11	359	401	760
	(1.7)	(0.6)	(0.4)	(0.5)	(16.3)	(12.6)	(14.0)	(0.5)	(0.2)	(0.3)	(27.9)	(19.7)	(22.9)
Taif	3311	9	52	61	130	261	391	41	38	79	302	488	790
	(1.7)	(0.7)	(2.7)	(1.8)	(9.5)	(13.5)	(11.8)	(3.0)	(2.0)	(2.4)	(22.0)	(25.2)	(23.9)
Bisha	2297	29	35	64	199	273	472	41	46	87	306	380	686
	(1.2)	(2.5)	(3.1)	(2.8)	(17.3)	(23.9)	(20.5)	(3.6)	(4.0)	(3.8)	(26.5)	(33.2)	(29.9)
Gurayyat	1266	0	0	0	107	114	221	2	0	2	151	140	291
	(0.7)	(0.0)	(0.0)	(0.0)	(16.7)	(18.2)	(17.5)	(0.3)	(0.0)	(0.2)	(23.6)	(22.3)	(23.0)
Total	193715	1424	1358	2782	11932	12476	24408	1054	1103	2157	19318	20564	39882
	(100)	(1.5)	(1.4)	(1.4)	(12.7)	(12.5)	(12.6)	(1.1)	(1.1)	(1.1)	(20.6)	(20.6)	(20.6)

Values are presented as a number and precentage (%).

Among the total screened older people, 8.5% were smokers, 55.7% regularly used their prescribed medications, and 25.3% of participants used more than 5 medications (polypharmacy). Jouf had the highest number of smokers (16.9%), polypharmacy was the highest in Jeddah (58.5%), while Konfothah reported the highest rate of taking medications regularly (Table 5).

**Table 5:** - Region-wise distribution of some health characteristics of the studied group during the period 2012-2020.

Health regions	All screened	Polypharmacy			Regular use of medication			Smoking		
		Male	Female	Total	Male	Female	Total	Male	Female	Total
Asir	27643	2318	2329	4647	7279	6341	13620	669	5	674
	(14.3)	(16.1)	(17.5)	(16.8)	(50.7)	(47.8)	(49.3)	(4.7)	(0.0)	(2.4)
Gassim	25354	3528	3196	6724	8712	7495	16207	1699	12	1711
	(13.1)	(26.2)	(26.8)	(26.5)	(64.8)	(62.9)	(63.9)	(12.6)	(0.1)	(6.7)
Baha	25450	3978	4175	8153	7424	7804	15228	2513	16	2529
	(13.1)	(31.8)	(32.3)	(32.0)	(59.3)	(60.4)	(59.8)	(20.1)	(0.1)	(9.9)
Jazan	21281	965	1724	2689	3585	5499	9084	1567	400	1967
	(11.0)	(10.6)	(14.2)	(12.6)	(39.4)	(45.2)	(42.7)	(17.2)	(3.3)	(9.2)
Riyadh	20667	2755	2806	5561	5718	5735	11453	1858	39	1897
	(10.7)	(26.6)	(27.2)	(26.9)	(55.3)	(55.6)	(55.4)	(18.0)	(0.4)	(9.2)
Eastern region	12588	2162	2318	4480	3146	3318	6464	1002	396	1398
	(6.5)	(35.4)	(35.8)	(35.6)	(51.5)	(51.2)	(51.4)	(16.4)	(6.1)	(11.1)
Hafer Albatin	7992	521	545	1066	2298	2429	4727	1191	150	1341
	(4.1)	(15.8)	(11.6)	(13.3)	(69.7)	(51.7)	(59.1)	(36.1)	(3.2)	(16.8)
Jeddah	7748	2430	2099	4529	2308	1895	4203	757	149	906
	(4.0)	(56.1)	(61.4)	(58.5)	(53.3)	(55.5)	(54.2)	(17.5)	(4.4)	(11.7)
Hail	6649	769	958	1727	1719	2049	3768	396	0	396
	(3.4)	(25.4)	(26.5)	(26.0)	(56.7)	(56.6)	(56.7)	(13.1)	(0.0)	(6.0)
Nagran	5415	418	519	937	1154	1735	2889	226	14	240
	(2.8)	(15.9)	(18.6)	(17.3)	(44.0)	(62.1)	(53.4)	(8.6)	(0.5)	(4.4)
Tabuk	4453	514	596	1110	1117	1536	2653	561	28	589
	(2.3)	(24.2)	(25.6)	(24.9)	(52.6)	(65.9)	(59.6)	(26.4)	(1.2)	(13.2)
Alahsaa	3921	790	690	1480	1304	1035	2339	389	1	390
	(2.0)	(38.6)	(36.8)	(37.7)	(63.7)	(55.3)	(59.7)	(19.0)	(0.1)	(9.9)
Almadinah	3696	370	1055	1425	603	2075	2678	92	28	120
	(1.9)	(39.7)	(38.2)	(38.6)	(64.6)	(75.1)	(72.5)	(9.9)	(1.0)	(3.2)
Jouf	3678	296	355	651	1205	1057	2262	571	51	622
	(1.9)	(15.4)	(20.2)	(17.7)	(62.7)	(60.2)	(61.5)	(29.7)	(2.9)	(16.9)
Konfothah	3627	418	601	1019	1291	1895	3186	413	11	424
	(1.9)	(28.6)	(27.8)	(28.1)	(88.2)	(87.6)	(87.8)	(28.2)	(0.5)	(11.7)
Makkah	3359	400	278	678	500	652	1152	311	88	399
	(1.7)	(22.8)	(17.3)	(20.2)	(28.5)	(40.6)	(34.3)	(17.7)	(5.5)	(11.9)
Northern borders	3320	305	456	761	960	1512	2472	332	72	404
	(1.7)	(23.7)	(22.5)	(22.9)	(74.5)	(74.4)	(74.5)	(25.8)	(3.5)	(12.2)
Taif	3311	212	229	441	630	809	1439	227	5	232
	(1.7)	(15.4)	(11.8)	(13.3)	(45.9)	(41.8)	(43.5)	(16.5)	(0.3)	(7.0)
Bisha	2297	348	285	633	637	643	1280	69	0	69
	(1.2)	(30.2)	(24.9)	(27.6)	(55.2)	(56.2)	(55.7)	(6.0)	(0.0)	(3.0)
Gurayyat	1266	148	136	284	421	362	783	75	7	82
	(0.7)	(23.2)	(21.7)	(22.4)	(65.9)	(57.7)	(61.8)	(11.7)	(1.1)	(6.5)
Total	193715	23645	25350	48995	52011	55876	107887	14918	1472	16390
	(100)	(25.2)	(25.4)	(25.3)	(55.4)	(56.0)	(55.7)	(15.9)	(1.5)	(8.5)

Values are presented as a number and precentage (%).

## Discussion

In the present study, a high prevalence of the studied chronic diseases DM, HTN, DM and HTN co-morbidity, and obesity was reported among the screened Saudi older people. In addition, the prevalence of special sense disorders (vision and hearing impairment) was also high, while the prevalence of positive screening results of the studied geriatric giants (depression, memory and cognitive impairment, risk of fall, and urine incontinence) was found to be less frequent. Moreover, there was a high prevalence of other risk factors like smoking and polypharmacy. The health regions varied widely in the prevalence of the studied chronic diseases and geriatric health conditions, and females reported a higher prevalence of most of the studied conditions.

In the current study, the prevalence of DM was 55.4%, this percentage is slightly higher than the Saudi Health Information Survey (SHIS),^19^ which reported a DM prevalence of 50.4% among those aged 65 years and above but slightly lower than the estimated DM prevalence by Al-Modeer et al^20^ who reported it to be 57.3%. Conversely, Khoja et al^5^ reported a lower prevalence of 32%, and when the use of anti-diabetic medication was accounted for, the prevalence of DM increased to 47%.

The prevalence of HTN was found to be 49.1%. A higher rate (59.1%) was reported by Al Modeer et al^20^ and by SHIS (51.2% among those aged 55-64 years and 70% among those aged 65 years and above).^19^ According to the household health survey report, the rate of high blood pressure increases with increasing age, gradually before the age of 40 years, and then rises sharply at 40 years and above, and it is noted that the percentage of high blood pressure diagnosed in the age group 65 years and over is the highest for both genders. The prevalence in this category is 54.5% among females, compared to 44.4% among males.^21^ However, lower prevalence of 30% was reported by Khoja et al^5^ and when antihypertensive medications were accounted for in estimating the prevalence of HTN, the prevalence increased to 42%.

In the present study, the prevalence of both DM and HTN among females (27.6%) was higher than males (25.9%). A higher prevalence of diagnosed chronic diseases was seen in the age group 65 years and over, and consisted of 75.8% females compared to 66.3% males.^21^ This can be explained by the fact that this study involved participants in the age group 60 and above while the household survey figures are related to those 65 years and above.

In the current study, the overall prevalence of underweight (BMI<18.5 kg/m^2^) was 5.4% (5.2% among females and 5.6% among males). This is lower compared to a cross-sectional study carried out among 38 females aged ≥60 years who were residents at the social welfare home for elderly females in Riyadh, Saudi Arabia, which reported 21% of the participants to be underweight.^22^ Similarly, another Saudi cross-sectional descriptive study carried out in PHCCs in Riyadh, Saudi Arabia, among 2045 older adults aged ≥60, reported the prevalence of malnutrition to be 20.9%.^23^ This difference may be related to the target population, sample size, and the location of the study.

Regarding the overall prevalence of anemia, in the current study using the WHO criteria, it was 4.7% with a higher rate among females (5.5%) than among males (3.9%). This finding differs from previous studies carried out in other countries, for instance, in the United States of America, using the same criteria, the prevalence of anemia in the elderly was found to range from 8-44%, with the highest prevalence in men aged 85 years and older.^24^ As anemia is a common condition in adults aged 60 years and older, and given the demographic growth of this population and the morbidity and mortality associated with anemia, primary care physicians should be familiar with the evaluation and management of anemia in older people.^25^

Regarding the prevalence of physician-diagnosed asthma among the studied group, it was found to be 8.9%. A higher physician-diagnosed asthma prevalence of 10.9% was observed in a nationwide, population-based survey of individuals aged ≥65 years, living in mainland Portugal.^26^

The current study found that 8.5% of older people were smokers. This rate is lower than that reported by SHIS (12.2%) among older people aged 65 years and above.^19^ These differences may be related to the age of the target population, sample size, socioeconomic status, culture, and lifestyle factors.

In the current study, the overall prevalence of positive screening for depression was 5.9%, ranging regionally between 2.1-18.9%. On the other hand, there was variation in depression prevalence reported by other studies carried out in Saudi Arabia. A study in 2021 that used PHQ-9 to assess the prevalence of depression among the geriatric population visiting PHCCs in the eastern region found that the prevalence was up to 42%.^27^ Furthermore, a 2017 study reported that 17% of the hospitalized patients were diagnosed with a major depressive disorder and 10.5% with other depressive disorders.^28^ The differences in the prevalence of depression among older people could be attributed to the target population, location of the study, social and cultural differences, and the use of different screening tools.

In the current study, the overall prevalence of those screened positive for cognitive impairment was 2.9%, with a wide range among the health regions (0.2-10.7%). Other studies in Saudi Arabia reported a more common prevalence. A cross-sectional multistage study that involved 1299 older individuals attending PHCCs in Riyadh between January 2015 and April 2017, using the Arabic version of the Mini-Mental State Examination (MMSE); found that 21% of the studied population had cognitive impairment.^29^ Furthermore, a community-based study in 2018 among 170 persons aged ≥60 years using the Arabic version of the Montreal Cognitive Assessment (MoCA) test reported the prevalence of cognitive impairment to be 45%, mild cognitive impairment to be 38.6%, and dementia at 6.4%.^30^ In addition, a recent study in Portugal that assessed the prevalence and incidence of cognitive impairment in the elderly population (65-85 years old) reported the prevalence of cognitive impairment to be 15.5%.^31^ These dissimilarities may be attributed to the differences related to age, language and education level of the studied group, sample size, and differences in the screening tool used, and cut-off scores for cognitive impairment. Therefore, for future studies, the homogenization of the definition of cognitive impairment and standardized cut-off scores of cognitive tests to compare different studies were proposed.^32^ In this study it was observed that the prevalence of DM, HTN, both DM and HTN co-morbidity, and cognitive impairment positive screening results was the highest among participants from Al-Ahsa region. This may be an interesting finding that warrants further studies.

In this study, the overall risk of fall among the studied older people was 4%, with a wide range among the regions (1.3-24.5%). Higher rates were observed by other Saudi studies such as a study in Unaizah, Qassim, Saudi Arabia, that reported it as 31.5% among 280 elderly patients aged >60 years old attending 10 randomly selected PHCCs during the period between January and October 2019.^33^ Similarly, higher prevalence of falls (49.9%) among the elderly was reported in a previous study carried out in Riyadh.^34^ The lower risk of fall rate in the current study compared to other studies may be attributed to the difference in the assessment tools, the study duration, and the target population number. The annual prevalence of falls has increased by age, from 28-35% for people aged ≥65 years to 32-42% for those aged >70 years. The frequency of falls increases with age and frailty level.^35^

Regarding the prevalence of urine incontinence in this study, it was 6.3%. Regionally, the prevalence range was 2.2-10.6%. A higher rate (41.4%) was reported by another Saudi study.^36^ In Mexico, the prevalence of urine incontinence was 9.5%, this rate is within the range of the regions observed in this study.^37^

In the present study, the prevalence of hearing impairment among the studied group was 12.6% and deafness was 1.4%. This is lower than that observed in a Saudi study by Al Rubeaan et al^38^ among patients with type 2 DM where 49% of patients had hearing loss in both ears, 8.3% in the right ear only, 8.9% in the left ear only, and 29% had disabling hearing loss.

In this study, the prevalence of vision impairment was 20.6% and blindness was 1.1%. The results of the current study are near to those reported by a previous Saudi population-based cross-sectional study carried out among 705 adults aged 18 years and older in Arar, Saudi Arabia, where 166 (23.5%) cases were found to have vision impairment while only 12 (1.7%) cases were considered as blind.^39^

In the current study, the overall prevalence of polypharmacy among the studied older people was 25.3%. This result is lower than that reported by an earlier Saudi retrospective cross-sectional study to evaluate the utilization of medications and comorbidities among 3009 geriatric patients (65 years and older) in the Prince Sultan Military Medical City, Riyadh, Saudi Arabia, database in 2018 which found that 55% of the patients had polypharmacy.^40^ This high prevalence of polypharmacy was explained by the fact that the elderly group admitted to the hospital were expected to have multiple comorbidities, which could lead to higher utilization and medication consumption.

### Study limitation

Statistical significance tests could not be carried out since we analyzed retrospective pooled data and could not compare the means of the males and females or the regions.

In conclusion, the study findings highlight the importance of CGA in the early detection of geriatric giants, common health problems, and associated risk factors among Saudi older people, which facilitate early intervention and management of any detected disorder to maintain and improve their health and quality of life. The current study indicates that the regions varied in the number of total people aged 60 years and above serviced by the program and delivered CGA. In addition, the regions had a wide range of prevalence of chronic diseases, geriatric giants, and health conditions. This was reflected in reporting a low mean prevalence of some of these conditions such as depression, memory and cognitive impairment, risk of fall, urine incontinence, and impairments of vision and hearing. The variation in the prevalence of chronic diseases among the regions was also reported by other Saudi health reports.^21^ Future in-depth clinical studies are needed to investigate the geriatric health conditions utilizing homogenous definitions, and screening and diagnostic tools and cut-off points. The impact of socio-demographic and biological risk factors on older people’s health should be thoroughly explored. A detailed national registry for older people health data is warranted.

## References

[1] United Nations. World population ageing 2020 highlights. [Updated 2020; accessed 2022 May 5]. Available from: https://www.un.org/development/desa/pd/sites/www.un.org.development.desa.pd/files/files/documents/2020/Sep/un_pop_2020_pf_ageing_10_key_messages.pdf

[2] World Health Organization. Fact sheet, ageing and health 2021. [Updated 2020; accessed 2022 May 5].Available from: https://www.who.int/news-room/fact-sheets/detail/ageing-and-health

[3] World Bank. World Databank: world development indicators (1960-2014) 2014. [Updated 2020; accessed 2022 May 5]. Available from: http://databank.worldbank.org/data

[4] United Nations. World population prospects: the 2012 revision 2012. [Updated 2020; accessed 2022 May 5]. Available from: https://data.un.org/Default.aspx

[5] Khoja AT , Aljawadi MH , Al-Shammari SA , Mohamed AG , Al-Manaa HA , Morlock L , et al. The health of Saudi older adults; results from the Saudi National Survey for Elderly Health (SNSEH) 2006-2015. Saudi Pharm J 2018; 26: 292-300. 30166931 10.1016/j.jsps.2017.11.008 PMC6111452

[6] Raina P , Gilsing A , Mayhew AJ , Sohel N , van den Heuvel E , Griffith LE. Individual and population level impact of chronic conditions on functional disability in older adults. PLoS One 2020; 15: e0229160. 32078637 10.1371/journal.pone.0229160 PMC7032687

[7] Mobbs C. The Merck manual of geriatrics. [Updated 2019; accessed 2022 May 9]. Available from: http://www.merck.com/pubs/mm_geriatrics/sec2/ch2.html

[8] Weber P , Meluzinova H , Matejovska-Kubesova H , Polcarova V , Jarkovsky J , Bielakova K , et al. Geriatric giants--contemporary occurrence in 12,210 in-patients. Bratisl Lek Listy 2015; 116: 408-416. 26286242 10.4149/bll_2015_078

[9] Ellis G , Gardner M , Tsiachristas A , Langhorne P , Burke O , Harwood RH , et al. Comprehensive geriatric assessment for older adults admitted to hospital. Cochrane Database Syst Rev 2017; 9: CD006211. 28898390 10.1002/14651858.CD006211.pub3 PMC6484374

[10] Chen Z , Ding Z , Chen C , Sun Y , Jiang Y , Liu F , et al. Effectiveness of comprehensive geriatric assessment intervention on quality of life, caregiver burden and length of hospital stay: a systematic review and meta-analysis of randomised controlled trials. BMC Geriatr 2021; 21: 377. 34154560 10.1186/s12877-021-02319-2 PMC8218512

[11] Ministry of Health. The national strategy for elderly in Saudi Arabia 2010-2015. [Updated 2020; accessed 2022 May 12]. Available from: https://extranet.who.int/countryplanningcycles/sites/default/files/planning_cycle_repository/saudi_arabia/lstrtyjy_lwtny_sh_lmsnyn_fy_lmmlk_lrby_lswdy_2015-2010-_national_strategy_health_of_the_elderly_in_saudi_arabia_2010-2015.pdf

[12] Ministry of health. Guideline for the Elderly Care Program in Health Centers. Second edition 2014 [in Arabic]. [Updated 2020; accessed 2022 May 5]. Available from: https://www.moh.gov.sa/Ministry/About/Health%20Policies/025.pdf

[13] World Health Organization (WHO). Age-friendly Primary Health Care Centers toolkit. [Updated 2008; accessed 2022 May 16]. Available from: https://extranet.who.int/agefriendlyworld/wp-content/uploads/2014/06/WHO-Age-friendly-Primary-Health-Care-toolkit.pdf

[14] MiniCog. Arabic mini-cog for Saudi Ministry of Health. [Updated 2020; accessed 2022 May 18]. Available from: https://mini-cog.com/about/using-the-mini-cog/

[15] Borson S , Scanlan JM , Watanabe J , Tu SP , Lessig M. Improving identification of cognitive impairment in primary care. Int J Geriatr Psychiatry 2006; 21: 349-355. 16534774 10.1002/gps.1470

[16] Park SH. Tools for assessing fall risk in the elderly: a systematic review and meta-analysis. Aging Clin Exp Res 2018; 30: 1-16. 28374345 10.1007/s40520-017-0749-0

[17] World Health Organization. Physical status: the use of and interpretation of anthropometry, report of a WHO expert committee. [Updated 1995; accessed 2022 May 20]. Available from: https://apps.who.int/iris/bitstream/handle/10665/37003/W?sequence=1 8594834

[18] Nutritional anaemias. Report of a WHO scientific group. World Health Organ Tech Rep Ser 1968; 405: 5-37. 4975372

[19] Ministry of Health. Saudi health information survey for non-communicable diseases in Kingdom of Saudi Arabia, 2013. [Updated 2020; accessed 2022 July 12]. Available from: https://www.moh.gov.sa/en/Ministry/Statistics/Pages/healthinformatics.aspx

[20] Al-Modeer MA , Hassanien NS , Jabloun CM. Profile of morbidity among elderly at home health care service in Southern Saudi Arabia. J Family Community Med 2013; 20: 53-57. 23723732 10.4103/2230-8229.108187 PMC3663165

[21] General Authority for Statistics. Household health survey report 2018. [Updated 2020; accessed 2022 July 14]. Available from: https://www.stats.gov.sa/sites/default/files/household_health_survey_2018.pdf

[22] Al Turki M , Al Sibaie N , Al Otaibi H , Al Ammari G , Al Otaibi R. Prevalence of geriatric malnutrition in long term care center in Riyadh/Saudi Arabia: a cross sectional study. ASNH 2021; 5: 11-17.

[23] Alhamdan AA , Bindawas SM , Alshammari SA , Al-Amoud MM , Al-Orf SM , Al-Muammar MN , et al. Prevalence of malnutrition and its association with activities of daily living in older adults attending primary health care centers: a multistage cross-sectional study. Progr Nutr 2019; 21: 1011-1018.

[24] Smith DL. Anemia in the elderly. Am Fam Physician 2000; 62: 1565-1572. 11037074

[25] Lanier JB , Park JJ , Callahan RC. Anemia in older adults. Am Fam Physician 2018; 98: 437-442. 30252420

[26] Pite H , Pereira AM , Morais-Almeida M , Nunes C , Bousquet J , Fonseca JA. Prevalence of asthma and its association with rhinitis in the elderly. Respir Med 2014; 108: 1117-1126. 24877896 10.1016/j.rmed.2014.05.002

[27] Alabdulgader A , Mobarki AO , AlDuwayrij A , Albadran A , Almulhim MI , Almulhim A. Depression screening for the geriatric population visiting primary healthcare centers in the Eastern region of Saudi Arabia. Cureus 2021; 13: e17971. 34667660 10.7759/cureus.17971 PMC8516422

[28] Alamri SH , Bari AI , Ali AT. Depression and associated factors in hospitalized elderly: a cross-sectional study in a Saudi teaching hospital. Ann Saudi Med 2017; 37: 122-129. 28377541 10.5144/0256-4947.2017.122 PMC6150550

[29] Alshammari SA , Alhamdan AA , Bindawas SM , Al-Amoud MM , Al-Orf SM , Al-Muammar MN , et al. Assessing the cognitive status of older adults attending primary healthcare centers in Saudi Arabia using the Mini-Mental State Examination. Saudi Med J 2020; 41: 1315-1323. 33294888 10.15537/smj.2020.12.25576 PMC7841595

[30] Alkhunizan M , Alkhenizan A , Basudan L. Prevalence of mild cognitive impairment and dementia in Saudi Arabia: a community-based study. Dement Geriatr Cogn Dis Extra 2018; 8: 98-103. 29706986 10.1159/000487231 PMC5921184

[31] Pais R , Ruano L , Moreira C , Carvalho OP , Barros H. Prevalence and incidence of cognitive impairment in an elder Portuguese population (65-85 years old). BMC Geriatr 2020; 20: 470. 33198643 10.1186/s12877-020-01863-7 PMC7667782

[32] Pais R , Ruano L , P Carvalho O , Barros H. Global cognitive impairment prevalence and incidence in community dwelling older adults-a systematic review. Geriatrics (Basel) 2020; 5: 84. 33121002 10.3390/geriatrics5040084 PMC7709591

[33] Alabdullgader A , Rabbani U. Prevalence and risk factors of falls among the elderly in Unaizah city, Saudi Arabia. Sultan Qaboos Univ Med J 2021; 21: e86-e93. 33777428 10.18295/squmj.2021.21.01.012 PMC7968899

[34] Almegbel FY , Alotaibi IM , Alhusain FA , Masuadi EM , Al Sulami SL , Aloushan AF , et al. Period prevalence, risk factors and consequent injuries of falling among the Saudi elderly living in Riyadh, Saudi Arabia: a cross-sectional study. BMJ Open 2018; 8: e019063. 10.1136/bmjopen-2017-019063 PMC5781015 29326189

[35] World Health Organization. Global report on falls prevention in older age 2007. [Updated 2020; accessed 2022 July 19]. Available from: http://www.who.int/ageing/publications/Falls_prevention7March.pdf

[36] Al-Badr A , Brasha H , Al-Raddadi R , Noorwali F , Ross S. Prevalence of urinary incontinence among Saudi women. Int J Gynaecol Obstet 2012; 117: 160-163. 22356760 10.1016/j.ijgo.2011.12.014

[37] Townsend MK , Lajous M , Medina-Campos RH , Catzin-Kuhlmann A , López-Ridaura R , Rice MS. Risk factors for urinary incontinence among postmenopausal Mexican women. Int Urogynecol J 2017; 28: 769-776. 27987024 10.1007/s00192-016-3196-0

[38] Al-Rubeaan K , AlMomani M , AlGethami AK , Darandari J , Alsalhi A , AlNaqeeb D , et al. Hearing loss among patients with type 2 diabetes mellitus: a cross-sectional study. Ann Saudi Med 2021; 41: 171-178. 34085541 10.5144/0256-4947.2021.171 PMC8176373

[39] Parrey MU , Alswelmi FK. Prevalence and causes of visual impairment among Saudi adults. Pak J Med Sci 2017; 33: 167-171. 28367193 10.12669/pjms.331.11871 PMC5368301

[40] Alsuwaidan A , Almedlej N , Alsabti S , Daftardar O , Al Deaji F , Al Amri A , et al. A Comprehensive overview of polypharmacy in elderly patients in Saudi Arabia. Geriatrics (Basel) 2019; 4: 36. 31096616 10.3390/geriatrics4020036 PMC6631642

